# Regulation of Pain Genes—Capsaicin vs Resiniferatoxin: Reassessment of Transcriptomic Data

**DOI:** 10.3389/fphar.2020.551786

**Published:** 2020-10-29

**Authors:** Rajeev K. Singla, Adiba Sultana, Md. Shahin Alam, Bairong Shen

**Affiliations:** ^1^ Institutes for Systems Genetics, Frontiers Science Center for Disease-related Molecular Network, West China Hospital, Sichuan University, Chengdu, China; ^2^ Center for Systems Biology, Soochow University, Suzhou, China

**Keywords:** neuromodulation, resiniferatoxin, capsaicin, DEGs (differentially expressed genes), TRPV1, neuropathic pain (NP)

## Abstract

Emerging evidence has shown a strong association between neuropathic pain and chronic diseases. In recent years, the treatment of neuropathic pain has attracted more attention. Natural products, such as capsaicin and resiniferatoxin, have been well utilized to treat this disease. In this study, we aim to compare the regulatory effects of capsaicin and resiniferatoxin on pain-related genes as well as on genes with no direct association with pain. Public transcriptomic and microarray data on gene expression in the dorsal root ganglia and genes associated with TRPV1 (+) neurons were obtained from the GEO database and then analyzed. Differentially expressed genes were selected for further functional analysis, including pathway enrichment, protein-protein interaction, and regulatory network analysis. Pain-associated genes were extracted with the reference of two pain gene databases and the effects of these two natural drugs on the pain-associated genes were measured. The results of our research indicate that as compared to capsaicin, resiniferatoxin (RTX) regulates more non pain-associated genes and has a negative impact on beneficial genes (off-targets) which are supposed to alleviate nociception and hypersensitivity by themselves. So, based on this study, we may conclude that capsaicin may be less potent when compared to RTX, but it will elicit considerably less adverse effects too. Thereby confirming that capsaicin could be used for the efficient alleviation of neuropathic pain with possibly fewer side effects.

## Introduction

Neuropathic pain is one of the most critical neurological diseases and disorders. It affects approximately 3.3 to 17.9% of the population (Cohen and Mao, 2014; He et al., 2020). A total of 14.7% of Chinese patients suffering from chronic pain in Hong Kong had neuropathic characteristics (Cheung et al., 2017). For the neuropathic and ischemic pain, neuromodulative treatment is a modern and effective mode of treatment (Sokal et al., 2011). Neuromodulation can be achieved by electrical or chemical methods, it aims to alter communication between nerves which can lead to an increase in pain threshold by modifying nociception, hypersensitivity, and analgesia (Richards et al., 2012). Traditionally, natural products were used for the treatment of various ailments, and now due to advancements in scientific research fields, natural products are being validated by scientific methods and are being used in the scientific treatment of various diseases and disorders (Scotti et al., 2016; Singla and Dubey, 2019; Singla et al., 2019). Researchers have also validated the role of natural products from terrestrial plants and marine sources, for the treatment of neuropathic pain (Alonso et al., 2003; Quintans et al., 2014).

Capsaicin, a well-known component obtained from various species of the Capsicum genus (chili peppers), is a very potent agent for the treatment of neuropathic pain. Apart for its potential in the treatment of neuropathic pain, capsaicin also acts as an anti-oxidant, anti-obesity, and is cardioprotective (Lu et al., 2020; Qiao et al., 2020). Its neuropathic mechanism is widely thought to be modulated through the TRPV1 channel (Caterina et al., 1997; Bais and Greenberg, 2020). After clinical level validation, the topical formulation of capsaicin is widely used and quite popular, specifically in the form of an 8% topical cutaneous patch (Baron et al., 2017; Anand et al., 2019), 0.075% topical cream (Derry et al., 2009), and 0.075% lotion (Kulkantrakorn et al., 2019).

Resiniferatoxin (RTX), a capsaicin analogue obtained from the cactus-like plant, *Euphorbia resinifera* (Hergenhahn et al., 1984), is 1,000 times more potent than capsaicin for the alleviation of neuropathic pain (Maggi et al., 1990). The mode of neuropathic pain alleviation in the case of RTX is also *via* the TRPV1 channel (Kissin and Szallasi, 2011). Sorreto Therapeutics, along with other organizations, are conducting clinical trials to establish the clinical efficacy of RTX in treating severe pain in cancer and knee osteoarthritis (Heiss et al., 2014; Hockman et al., 2018; Unger et al., 2019). The treatment of sensory neurons with capsaicin or RTX causes calcium cytotoxicity that rapidly sutures and selectively deletes TRPV1(+) neurons (Isensee et al., 2014).

Isensee and co-workers had studied the effect of capsaicin and RTX in the dorsal root ganglion of rats and submitted the gene expression data in the NCBI (GSE59727). In their research, though they had used and collected data on both capsaicin and RTX, in their manuscript, they mainly focused on the characterization of genes associated with TRPV1(+) neurons. In their published manuscript, they had not correlated and compared the gene regulation of capsaicin and RTX in detail. Very little information has been made available for readers on capsaicin. Further, to the best of our knowledge, there has been no detailed comparison on the effect of capsaicin and RTX on pain genes and non pain-associated genes (Isensee et al., 2014). As RTX is proposed to be highly potent when compared to the already established capsaicin, and the evidence collection for its use in cancer and knee osteoarthritis is in process, it is extremely important to look at both of the drugs in terms of proteomics and gene regulations. This will help in the assessment of the possible adverse effects on the long-term usage of the drugs.

So, in our present study, we have reassessed the transcriptomic data submitted by the team of Isensee and analyzed it in the perspective as mentioned.

## Materials and Methods

### Microarray Data

The raw data of GSE59727, including 12 tissues (4 tissues for DMSO, 4 tissues for capsaicin, and 4 tissues for RTX), were obtained from the GEO database (https://www.ncbi.nlm.nih.gov/geo/), using the key words “capsaicin” AND “Rattus norvegicus” [porgn::txid10116] AND “neuron” and “tissues” (attribute name). These data were based on the GPL6101 platforms (Illumina ratRef-12 v1.0 expression beadchip) contributed to by Isensee and co-workers (Isensee et al., 2014). Four replicate experiments with RNA from the dorsal root ganglia (DRG) neurons of one rat per experiment were performed. Segregated neurons were split up into three parts, treated with solvent DMSO (0.1%), capsaicin (10 µM), or RTX (100 nM), and were followed by gradient centrifugation for 30 min (Isensee et al., 2014).

### Data Preprocessing and Differentially Expressed Gene Identification

The R package “limma V3.40.6” was used to identify DEGs in rat tissues compared with corresponding tissues treated with DMSO (0.1%) and capsaicin (10μM) or DMSO (0.1%) and RTX (100nM). We used log2-transformation to normalized the data, after that we used the normalized data for further analysis. We used the *t*-test and Benjamini–Hochberg methods to calculate the *p*-value and adjusted *the p*-value and logFC accordingly. To get the differentially expressed genes we set the threshold value as; *p*-value < 0.01 and | logFC | > 0.5, in both cases.

### Enrichment Analysis

ShinyGO is a graphical web application that displays the functional acuteness from a set of genes; which was developed under different R/Bioconductor packages. In this study, we use ShinyGO (http://bioinformatics.sdstate.edu/go/) to perform enrichment analysis (Ge et al., 2020). For the enrichment analysis we set the cutoff *p*-value = 0.05.

### Analysis of the Protein–Protein Interaction (PPI) Network of the DEGs

We used the Search Tool for the Retrieval of Interacting Genes (STRING v11.0) database (http://string-db.org) for constructing the PPI network of DEGs both from capsaicin and RTX-treated conditions. Then, the Cytoscape software (version 3.8.0) with the MCODE and Networkanalyzer app was used to display the PPI networks between the DEGs.

### Differentiation Between Pain-Associated Genes and Non-Pain Genes

The DEGs for capsaicin and RTX were then subjected to the Pain Networks database (http://www.painnetworks.org/rat/) for filtering of the pain-related genes with categorization with the Pain Gene Enrichment (PGE) score (Perkins et al., 2013). Further, DEGs for capsaicin and RTX were then subjected to the pain gene database (http://www.jbldesign.com/jmogil/PainGenedb_content.html) for more precise filtering of pain-related genes as the mentioned database also specifies the default function of the pain-related genes (Lacroix-Fralish et al., 2007).

## Results and Discussion

### Identification of Differentially Expressed Genes

**Figure 1:**
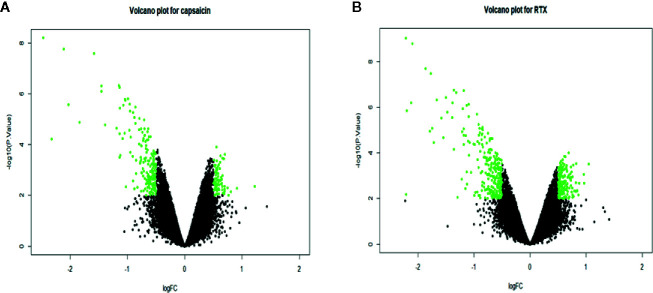
Volcano plot for representing the upregulated and downregulated genes in the capsaicin treatment group **(A)** and the RTX treatment group **(B)**.

**Figure 2:**
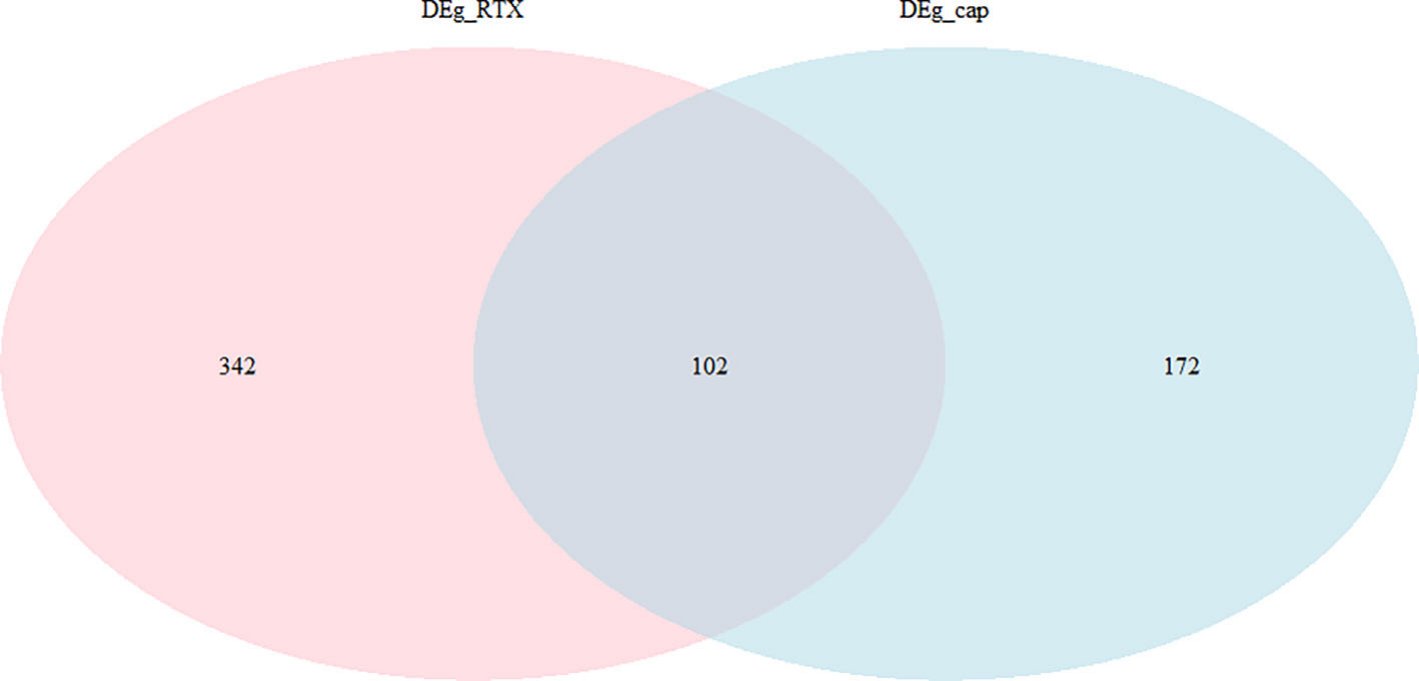
Overlapped genes in the capsaicin treatment group and the RTX treatment group.

We separated the datasets into two parts, one (capsaicin treatment group) is, DMSO (0.1%) and capsaicin (10µM); the other (RTX treatment group) is, DMSO (0.1%) and RTX (100nM). A total of 274 DEGs were identified, including 84 upregulated and 190 downregulated genes in the case of the capsaicin treatment group. But in the case of the RTX treatment group, a total of 444 DEGs were identified, including 171 upregulated and 273 downregulated genes (**Figure 1**). The top 30 upregulated and downregulated DEGs for capsaicin and RTX are displayed in **Tables S1** and **S2**, respectively. Further on the basis of the filters adopted, we found 102 genes which were common in both RTX and capsaicin (**Figure 2**).

### Enrichment Analysis

**Figure 3:**
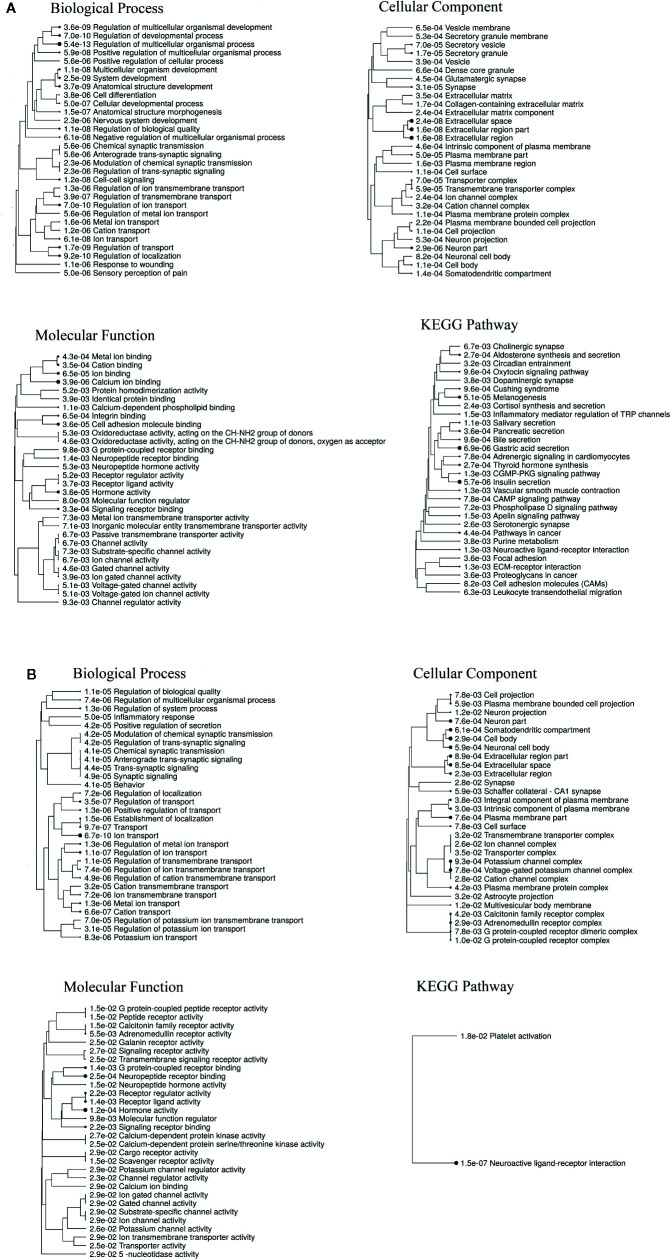
**(A)** Enrichment analysis of the DEGs for RTX. **(B)** Enrichment analysis of the DEGs for capsaicin.

Gene ontology (GO) enrichment analysis showed that DEGs for both RTX and capsaicin-treated conditions were significantly enriched in the biological process (BP), cellular component (CC), and molecular function (MF). Furthermore, KEGG pathway enrichment analysis indicated that DEGs were significantly enriched in 30 pathways (**Figure 3**). Both the RTX and capsaicin-treated genes share some GO (biological process) like ion transport, the regulation of ion transport, the regulation of transport, cation transport and the regulation of the multicellular organismal process. In the case of GO (cellular component), they share common cellular components like cell body, neuronal cell body, plasma membrane parts, neuron parts, and synapses. In GO (molecular function) enrichment, they share hormone activity, neuropeptide receptor binding, G protein-coupled receptor binding, molecular function regulators, and neuropeptide hormone activity. The KEGG pathways show that genes after treatment with both RTX and capsaicin are involved in neuronal functions, as well as other functions which are closely related with pain (**Figures 3A, B**). There is a KEGG pathway which is common to both the RTX and capsaicin-treated genes, like the neuroactive ligand-receptor interaction (**Figures 3A, B**). These genes appear in several signaling pathways that are specific to neurons, pain, and also to cellular machinery/organelles that are found in most cell types.

### Protein–Protein Interaction (PPI) Network of the DEGs

Based on the STRING online database for *Rattus norvegicus*, a total of 274 DEGs (84 upregulated and 190 downregulated genes) from capsaicin-treated conditions and 444 DEGs (171 upregulated and 273 downregulated genes) were filtered into the DEGs PPI network complex from RTX-treated conditions. Initially, in the case of capsaicin, the original number of nodes and edges was 183 and 164, respectively, where the expected number of edges was 59 (**Figure S1**). Hence the network had significantly more interactions than expected. Here the average node degree was 1.79 and the average local clustering coefficient was 0.363. But in order to have better protein-protein interaction analysis, this was further expanded to a level where the number of nodes and edges were 243 and 559, respectively, with the expected number of edges at 221. Average node degree was improved to 4.6 with an average local clustering coefficient of 0.484 (**Figure S2**). Similarly, in case of RTX, the original number of nodes and edges was 325 and 531, respectively, with an average node degree of 3.27 where the expected number of edges was 192, and the average local clustering coefficient was 0.36 (**Figure S3**). It was further expanded to a level where number of nodes and edges were 375 and 1,101, respectively, with an expected number of edges of 438. The average node degree was improved to 5.87 with an average local clustering coefficient of 0.425 (**Figure S4**). In both cases, the PPI enrichment *p*-value was less than 1.0e-16.

**Figure 4:**
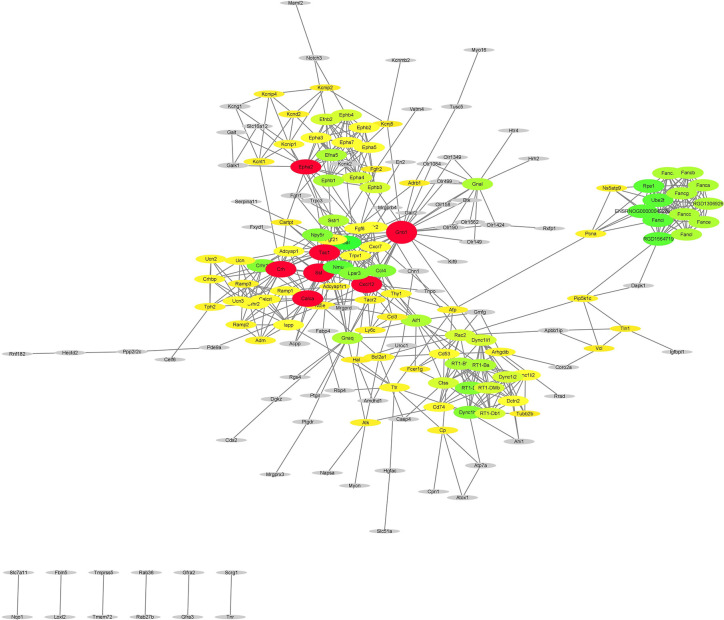
PPI network of the genes regulated after capsaicin treatment.

**Figure 5:**
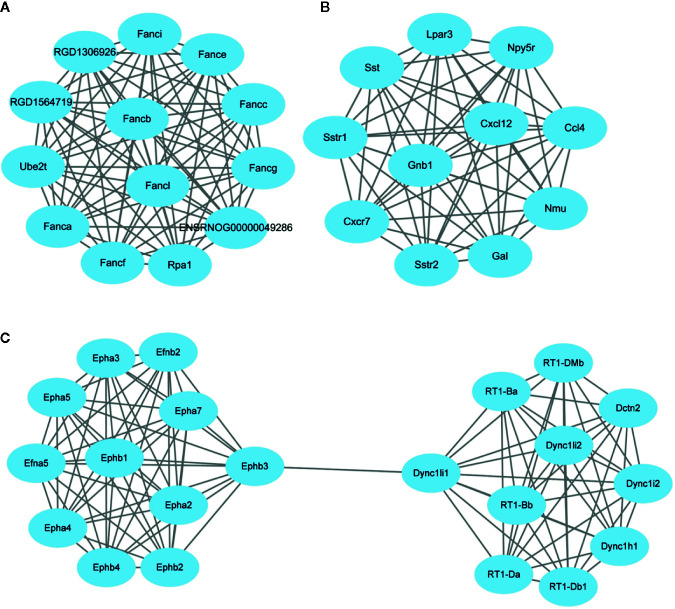
Clusters **(A–C)** of hub genes regulated after capsaicin treatment.

After subjecting the STRING dataset to Cytoscape 3.8.0, the protein-protein interactions were analyzed using the Networkanalyzer and MCODE application and different genes were categorized on the basis of degree of centrality (DoC). The genes having higher DoC have been represented both by bigger size as well as by different color for the ease of readers. In case of capsaicin, the color gradient is as follows: red for 15-27 DoC; green for 9.99-15 DoC; yellow for 3-9.99 DoC, and grey for below 3 DoC. The genes in the capsaicin-treated group with a higher DoC were Sst (upregulated), Crh (downregulated), and Tac1 (downregulated) (**Figure 4**). There were some more genes which were shown to have a higher DoC like Calca, Cxcl12, Gnb1, and Epha2, but they were not originally expressed by capsaicin. They were in fact the extended network genes by STRING. Other genes with a good DoC after capsaicin treatment were Gal (downregulated), Nmu (upregulated), RT1-Da (upregulated), Ccl4 (upregulated), Npy5R (downregulated), Lpar3 (downregulated), and Fancl (upregulated). Three main clusters of hub genes have also been identified using the MCODE app in the capsaicin-treated group (**Figure 5**). The fact that cluster B had 11 genes is of importance and some of the significant genes will be briefly discussed later.

**Figure 6:**
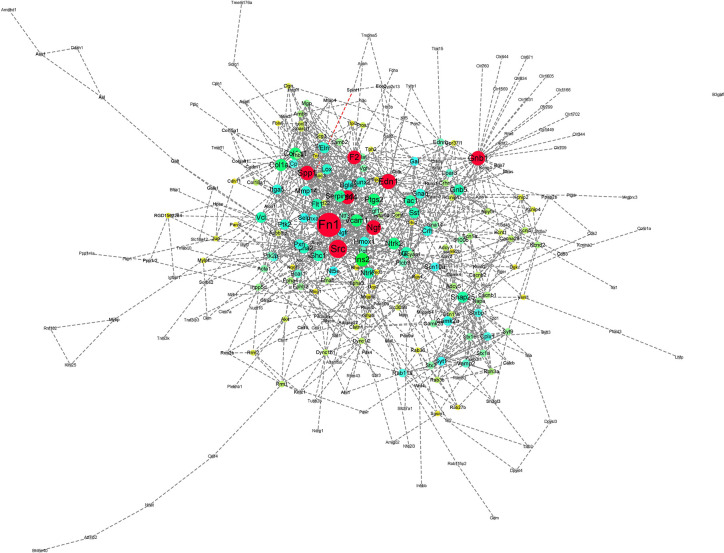
PPI network of the genes regulated after RTX treatment.

**Figure 7:**
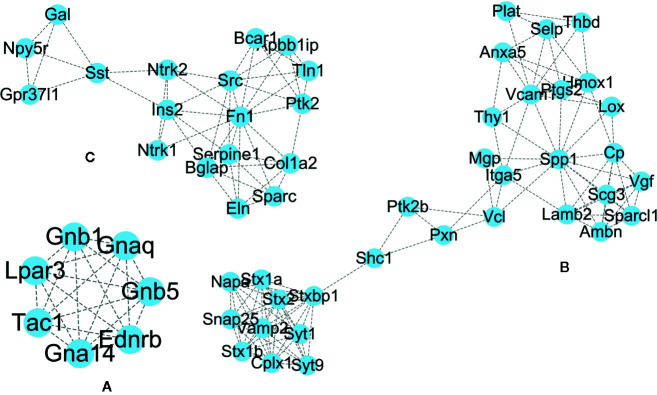
Clusters **(A–C)** of hub genes regulated after RTX treatment.

In the case of RTX, the color gradient is as follows: red for 29.78-61 DoC; green for 20-29.78 DoC; blue for 15.5-20 DoC; yellow to blue for 4.3-15.5 DoC; and grey for below 4.3 DoC. The genes in the RTX-treated group with a higher DoC were Fn1 (upregulated), Edn1 (upregulated), Spp1 (upregulated), and Cd44 (downregulated) (**Figure 6**). There were some more genes which were shown to have a higher DoC like Src, Ngf, Gnb1, and F2, but they were not the originally expressed by RTX. They were in fact extended network genes by STRING. Other genes with a good DoC after RTX treatment were Ins2 (downregulated), Col1a1 (upregulated), Vcam1 (upregulated), Ptgs2 (upregulated), Tac1 (downregulated), and Ntrk1 (downregulated). Three main clusters of hub genes have also been identified using the MCODE app in RTX-treated group (**Figure 7**). All these three clusters A-C are of importance and some of the significant genes from them will be briefly discussed later.

After the filtering of DEGs for capsaicin and RTX through the pain networks database, the DEGs were categorized by their PGE score. In the case of the capsaicin-treated group, out of 190 downregulated genes, there were only 42 genes with a PGE score ranging from 1-100. The genes with a PGE>20 were Gnaq, Tnr, Tac1, Gfra2, Acpp, Ptgir, Nt5e, Amigo2, Kcnip1, Adcyap1, Rgs4, Lpar3, Grik1, Kcnip2, Trpv1, Hrh2, Kcnj5, Gal, Plcl1, Kcnk2, and Iapp (*arranged in increasing order*). Out of 84 upregulated genes in the capsaicin-treated group, only 15 genes had a PGE score ranging from 1-100. The genes with a PGE>20 were Btk, Adrb1, Fcer1g, and Nmu (*arranged in increasing order*).

In the case of the RTX-treated group, out of 273 downregulated genes, there were only 75 genes with a PGE score ranging from 1-100. The genes with a PGE>20 were Camk2a, Gnaq, Plcb3, Comt, Tnr, Inpp5d, Ucp2, Tac1, Gfra2, Acpp, Adcy5, Ntrk1, Fgf13, Arhgap22, Nfe2l3, Ticam2, Nt5e, Amigo2, Adcyap1, Kcnip1, Cd22, Rgs4, Lpar3, Grik1, Kcnip2, Scn10a, Trpv1, Plcl1, Ece2, Scn11a, Hrh2, Kcnj5, Gal, Kcnk2, and Iapp (*arranged in increasing order*). Out of 171 upregulated genes in the RTX-treated group, only 43 genes had a PGE score ranging from 1-100. The genes with a PGE>20 were Sparc, Edn1, S100b, Itga5, Ptgs2, Sparcl1, Tyrp1, Fzd1, and Myl9 (*arranged in increasing order*). It is quite evident from the above data, that even at a low concentration, RTX is capable of regulating more pain-associated genes than capsaicin. But closely looking at the data, it also revealed that the effect of RTX is more non-specific when compared to capsaicin, because in the case of RTX, there are indeed more DEGs with no PGE score.

Further, filtering the DEGs through the Pain Genes database has its own significance as there are inherently few genes in our system which have been designed to alleviate pain, so the downregulation of such genes are actually not appropriate. As per filtering through this database, it has come to our notice that out of 274 DEGs in the capsaicin-treated group, only two upregulated and 21 downregulated DEGs had a direct influence on nociception, hypersensitivity, and analgesia (**Table 1**). On the other hand, in the case of the RTX-treated group, six upregulated and 31 downregulated DEGs had a direct influence on nociception, hypersensitivity, and analgesia (**Table 2**).

**Table 1:** Pain related genes regulated by capsaicin.

S.No.	Gene symbol	LogFC	Regulation	Nociception^1^	Hypersensitivity^1^	Analgesia^1^
1.	Ctss	0.646	Up	No effect	Increase	N.T
2.	Nmu	0.509	Up	Increase	N.T	N.T
3.	Iapp	-2.469	Down	Increase	N.T	N.T
4.	Lpar3	-1.834	Down	No effect	Increase	N.T
5.	Trpv1	-1.581	Down	Increase	Increase	N.T
6.	Kcnk2	-1.139	Down	Decrease	Decrease	Increase
7.	Acpp	-1.041	Down	No effect	Decrease	N.T
8.	Adcyap1	-0.991	Down	Increase	Increase	N.T
9.	Grik1	-0.903	Down	Increase	No Effect	N.T
10.	Rgs4	-0.861	Down	No effect	N.T	Increase
11.	Galr2	-0.740	Down	No effect	Contradictory data	N.T
12.	Kcnj5	-0.732	Down	Decrease	N.T	No effect
13.	Plcl1	-0.696	Down	Decrease	Decrease	N.T
14.	Gal	-0.684	Down	Decrease	Increase	N.T
15.	Nt5e	-0.680	Down	Decrease	Decrease	Increase
16.	Tac1	-0.648	Down	Increase	Contradictory data	Contradictory data
17.	Kcnt1	-0.639	Down	No effect	Decrease	N.T
18.	Hrh2	-0.606	Down	Increase	N.T	Increase
19.	Gnaq	-0.605	Down	Increase	Increase	N.T
20.	Gfra2	-0.595	Down	Decrease	N.T	N.T
21.	Ptgdr	-0.549	Down	Decrease	N.T	N.T
22.	Camk4	-0.536	Down	No effect	N.T	Increase
23.	Ptgir	-0.509	Down	Increase	Increase	N.T

^1^These are the default functions of the specified genes. The information is gathered from the pain gene database (Lacroix-Fralish et al., 2007). N.T, not tested. Red: undesired gene response; green: desired gene response; orange: off-targets for capsaicin.

**Table 2:** Pain related genes regulated by RTX.

S.No.	Gene symbol	LogFC	Regulation	Nociception^1^	Hypersensitivity^1^	Analgesia^1^
1.	Ptgs2	0.685	Up	Increase	Increase	N.T
2.	S100b	0.613	Up	N.T	Increase	N.T
3.	Spp1	0.550	Up	Increase	No effect	N.T
4.	Sparc	0.545	Up	Decrease	N.T	N.T
5.	Tyrp1	0.543	Up	Increase	N.T	N.T
6.	Edn1	0.534	Up	Decrease	Decrease	N.T
7.	Iapp	-3.536	Down	Increase	N.T	N.T
8.	Trpv1	-2.215	Down	Increase	Increase	N.T
9.	Lpar3	-2.198	Down	No effect	Increase	N.T
10.	Adcyap1	-1.864	Down	Increase	Increase	N.T
11.	Kcnk2	-1.663	Down	Decrease	Decrease	Increase
12.	Gal	-1.581	Down	Decrease	Increase	N.T
13.	Tac1	-1.503	Down	Increase	Contradictory data	Contradictory data
14.	Acpp	-1.386	Down	No effect	Decrease#009966	N.T
15.	Grik1	-1.318	Down	Increase	No effect	N.T
16.	Galr2	-1.202	Down	No effect	Contradictory data	N.T
17.	Rgs4	-1.187	Down	No effect	N.T	Increase
18.	Nt5e	-0.940	Down	Decrease	Decrease	Increase
19.	Kcnj5	-0.919	Down	Decrease#009966	N.T	No effect
20.	Kcnt1	-0.904	Down	No effect	Decrease	N.T
21.	Gfra2	-0.863	Down	Decrease	N.T	N.T
22.	Hrh2	-0.801	Down	Increase	N.T	Increase
23.	Camk2a	-0.790	Down	Increase	Increase	N.T
24.	Scn11a	-0.712	Down	Increase	Contradictory data	N.T
25.	Adcy5	-0.693	Down	Increase	Increase	Increase
26.	Cacna2d1	-0.669	Down	Increase	Increase	N.T
27.	Ptgdr	-0.647	Down	Decrease	N.T	N.T
28.	Gnaq	-0.635	Down	Increase	Increase	N.T
29.	Camk4	-0.628	Down	No effect	N.T	Increase
30.	Ucp2	-0.617	Down	No effect	N.T	Decrease
31.	Ntrk1	-0.567	Down	Increase	N.T	N.T
32.	Ece2	-0.545	Down	No effect	N.T	Increase
33.	Plcl1	-0.542	Down	Decrease	Decrease	N.T
34.	Runx1	-0.536	Down	Increase	Increase	N.T
35.	Comt	-0.533	Down	Decrease	N.T	Contradictory data
36.	Plcb3	-0.515	Down	No effect	N.T	Decrease
37.	Scn10a	-0.500	Down	Decrease	Contradictory data	Decrease

^1^These are the default functions of the specified genes. The information is gathered from pain gene database (Lacroix-Fralish et al., 2007). N.T, not tested. Red: undesired gene response; green: desired gene response; orange: off-targets for RTX.

After overlapping the data obtained from both the databases, there were some interesting observations. For instance, there are a few pain-related genes like Ctss, Galr2, Kcnt1, and Ptgdr, which have a zero PGE score, but from the pain gene database, it is very clear that these genes have a pain-related function. Further, after cross-validation with the original source manuscript of Isensee and co-workers (Isensee et al., 2014) whose transcriptomic data have been reprocessed by our team, it is also observable that there are a few pain-associated genes like S100b, Spp1, Sparc, Tyrp1, Edn1, Gfra2, Hrh2, Ucp2, Ntrk1, Ece2, Plcl1, Runx1, Comt, and Scn10a which have not been discussed by them in their published manuscript, or been provided as information in form of the supplementary data.

There are few indirect genes associated with pain. For instance, the Ina gene, which is downregulated by RTX (LogFC: -0.905) and capsaicin (LogFC: -0.795), is not directly a pain gene, but is associated with multiple genes which are associated with an increase in nociception like Cdk5, CdK5r1, Csk, Camk2a, Prkce, Pmp22, and Tyrp1 and decreases analgesia like Grk5, Prkce, and Prkcc. So, the downregulation of the Ina gene somehow affects these genes as well (Lacroix-Fralish et al., 2007).

The Hal gene, which is downregulated by capsaicin (LogFC: -0.665), is not a direct pain gene, but is associated with Kcnd2 which is linked with increased hypersensitivity, thus the downregulation of Hal gene somehow influences the effect of Kcnd2 (Lacroix-Fralish et al., 2007).

The Gnal gene, which is downregulated by capsaicin (LogFC: -0.500), is linked with genes like Ccr5, Slc12a5, Slc12a2, and Trpv1 which can increase nociception (Lacroix-Fralish et al., 2007).

The Plat gene, which is upregulated by RTX (LogFC: 0.536), is not directly a pain gene either, but it is associated with the Ptafr gene which is responsible for increased nociception and hypersensitivity. So upregulating the Plat gene will indirectly affects the Ptafr gene as well (Lacroix-Fralish et al., 2007).

Similarly, Lox is not a pain gene but it is upregulated by RTX (LogFC: 0.733), and is associated with many genes which are responsible for a decrease in nociception like Fstl1, Nedd4l, Crip2, Ret, Kcnq2, and Edn1 as well as a decrease in hypersensitivity like Nedd4l, Crip2, and Edn1 (Lacroix-Fralish et al., 2007).

While analyzing the data tabulated in **Tables 1** and **2**, we have segregated downregulated pain genes into desired/targets and undesired/off-targets. Here, desired targets means that their downregulation will decrease either nociception or hypersensitivity while undesired/off-targets means that those genes are already beneficial genes in alleviating nociception or hypersensitivity. Thus, the downregulation of off-targets here is somehow decreasing the innate capability of the host system to alleviate pain.

### Capsaicin Induced Upregulated Genes With No Direct Association With Pain Nociception

We will discuss here the biological functions of the genes which fall under the top 10 upregulated DEGs after capsaicin treatment (**Table S1** lists the top 30 upregulated and downregulated genes), without having any direct association with pain nociception. Olr1349 and Olr190 are genes that encode the olfactory receptor which is primarily responsible for the perception of smell (Gibbs et al., 2004; Antunes and Simoes De Souza, 2016). Tmprss5 which is a transmembrane serine protease 5 (also known as spinesin) is reported to be involved in the function of astrocytes in the spinal cord (Yamaguchi et al., 2008). Notch3 is regarded as the key gene for the maintenance and function of vascular smooth muscle cells, including those which are involved in the blood supply to the brain (Lin et al., 2019). But some of recent studies also suggest that Notch3 plays a vital role in oncogenesis, the maintenance of tumors, and chemotherapy resistance (Aburjania et al., 2018). Ccl4 is a monokine possessing chemokinetic and inflammatory properties and is suggested to be involved in neutrophil recruitment in the intrapulmonary region. It is also recorded as one of the major suppressive factors for HIV (Farquhar et al., 2005; Lee et al., 2007). The Car13 gene encodes carbonic anhydrases 13 (CA XIII) which is a cytosolic isoform of carbonic anhydrases. CA XIII, though it has a moderate catalytic activity, may contribute in maintaining the acid-base homeostasis in the kidneys, GI tract, and in the reproductive system (Hilvo et al., 2008). The Napsa gene encodes napsin A aspartic peptidase which is preferentially expressed in the kidneys, lungs, and spleen (Schauer-Vukasinovic et al., 2000). Its expression is commonly reported to be associated with pulmonary adenocarcinomas and renal cell carcinomas (Nam et al., 2016). The Colec12 gene is a collectin sub-family member 12, which encodes the scavenger receptor with the C-type lectin/carbohydrate recognition domain. These receptors are considered as an important member contributing to innate immunity (Nakamura et al., 2001). The Eif2a gene encodes eukaryotic translation initiation factor 2-alpha, which is generally observed as an environmental stress-induced gene, and phosphorylation of which results in the reduction of global protein synthesis (Hong et al., 2016). Though these genes may not have a direct association with pain nociception, most of these genes are found to influence nervous system functionality including inflammation.

### Capsaicin Induced Downregulated Genes With No Direct Association With Pain Nociception

We will discuss here the biological functions of the genes which fall under the top 10 downregulated DEGs after capsaicin treatment (**Table S1** lists the top 30 upregulated and downregulated genes), without having any direct association with pain nociception. The Cartpt gene encodes to the cocaine and amphetamine-regulated transcript (CART) peptide, which is evidently involved in the negative regulation of estradiol production (Lv et al., 2009), and is also involved in various stages of opioid addiction (Bakhtazad et al., 2016). The Mrgprx3 and Mrgprd genes encode for a G-protein coupled receptor (GPCR) for which adenine acts as the endogenous ligand and which plays a role in nociception, although its clear and specific role has not yet been defined (Bender et al., 2002). The Kcnip1 gene encodes for the potassium voltage gated ion channel, Kcnip1 which is primarily expressed in the brain and testes. Potassium channel blockers are used for the treatment of neuromuscular disorders, thus the downregulation of this gene seems to play an important role in the improvement of the nervous system (Del Pino et al., 2015). The Slc51a gene encodes for the organic solute transporter, and is involved in the transportation of bile acid as well as steroids in the intestine, renal, and biliary epithelia (Ballatori et al., 2005; Christian et al., 2012). The Amdhd1 gene encodes for the aminohydrolase domain which is involved in the formation of intestinal stem cells in adults (Okada et al., 2015). The Trpc3 gene encodes for member 3 of the subfamily C of the transient receptor potential cation channel which is a calcium-activated cation channel, and it is involved in the development of febrile seizures. Thus downregulation of the Trpc3 gene or inhibition of this calcium-activated cation channel significantly attenuates the susceptibility of seizures, neuronal cell death, and neuroinflammation (Sun et al., 2018).

### RTX Induced Upregulated Genes With No Direct Association With Pain Nociception

We will discuss here the biological functions of the genes which fall under the top 10 upregulated DEGs after RTX treatment (**Table S2** lists the top 30 upregulated and downregulated genes), without any direct association with pain nociception. As discussed above in the capsaicin section, the Notch3 gene is involved in the maintenance of vascular smooth muscle cells (Lin et al., 2019). Itga5, though it has a PGE score when filtered through the pain network database, no direct association was found when screened through the pain gene database. Itga5 encodes for the integrin subunit alpha 5, and is involved in the regulation of spine morphogenesis as well as the formation of synapses in hippocampal neurons (Webb et al., 2007). The Traf3ip3 gene encodes TRAF3 interacting protein 3 which modulates the pathway associated with c-Jun N-terminal kinase signal transduction, and thereby mediates cell growth (Dadgostar et al., 2003). Further, its upregulation has also been observed in various types of cancers (Nasarre et al., 2018). The Hpse gene is encodes for heparanase which is a endo-beta-glucouronidase and catalyzes the cleavage of heparan sulfate. Heparanase is evidently involved in neuroinflammatory responses (Changyaleket et al., 2017). The Mylip gene encodes the myosin regulatory light chain interacting protein which is involved in the Reelin-induced decrease of very low density lipoprotein receptors in neuronal systems (Do et al., 2013). The Pxn gene encodes to paxillin which is involved in the focal adhesion of cells with an extracellular matrix (Torres et al., 2008). Further, paxillin was reported to be involved in the regulation of cytoskeleton proteins like α-actin, α-tubulin, and destrin (Chen et al., 2014). The Aoc3 gene encodes for amine oxidase, copper containing 3, which is also recorded as vascular adhesion protein-1 and found to be involved in leucocyte recruitment (Noda et al., 2008). The Plpp3 gene encodes for the phospholipid phosphatase 3 enzyme and it is involved in the transport carrier’s formation in Golgi bodies (Gutierrez-Martinez et al., 2013). The Bace2 gene encodes for the beta-secretase 2 enzyme which is also recorded as aspartic protease and is involved in the cleavage of the precursor protein of amyloid-β into amyloid-β. Amyloid-β is well known to trigger neurodegenerative diseases like Alzheimer’s disease (Bettegazzi et al., 2011). The S100a16 gene encodes for S100 calcium-binding protein A16 which is involved in adipogenesis and thus weight gain (Zhang et al., 2014).

### RTX Induced Downregulated Genes With No Direct Association With Pain Nociception

We will discuss here the biological functions of the genes which fall under the top 10 downregulated DEGs after RTX treatment (**Table S2** lists the top 30 upregulated and downregulated genes), without any direct association with pain nociception. As already described in the relevant capsaicin section, the Cartpt gene is involved in various stages of opioid addiction (Bakhtazad et al., 2016). Though, the Crh gene does not have any association with pain, it possesses a good DoC (**Figure 6**) which indicates that it is closely associated with many other genes. Further, **Figure 4** represents the Crh gene in the capsaicin-treated protein-protein interaction, but in capsaicin, the downregulation of the Crh gene did not fall into the top 30 downregulated genes (**Table S1**). The Crh gene encodes the corticotrophin releasing hormone which is well known for its mediatory effect in the neuroendocrine stress response (Kozakai et al., 2019). The Mrgprx3 and Mrgprd genes which encode GPCR have recorded significance in nociception, though a specific role is as yet unclear (Bender et al., 2002). The Gfra3 gene encodes for the alpha 3 isoform of GDNF family receptor and is critically involved in neuronal cell protection (Hoke et al., 2000). The functionality of the Slc51a gene which encodes for the organic solute transporter has already been discussed in the capsaicin section (Ballatori et al., 2005; Christian et al., 2012).

**Figure 8:**
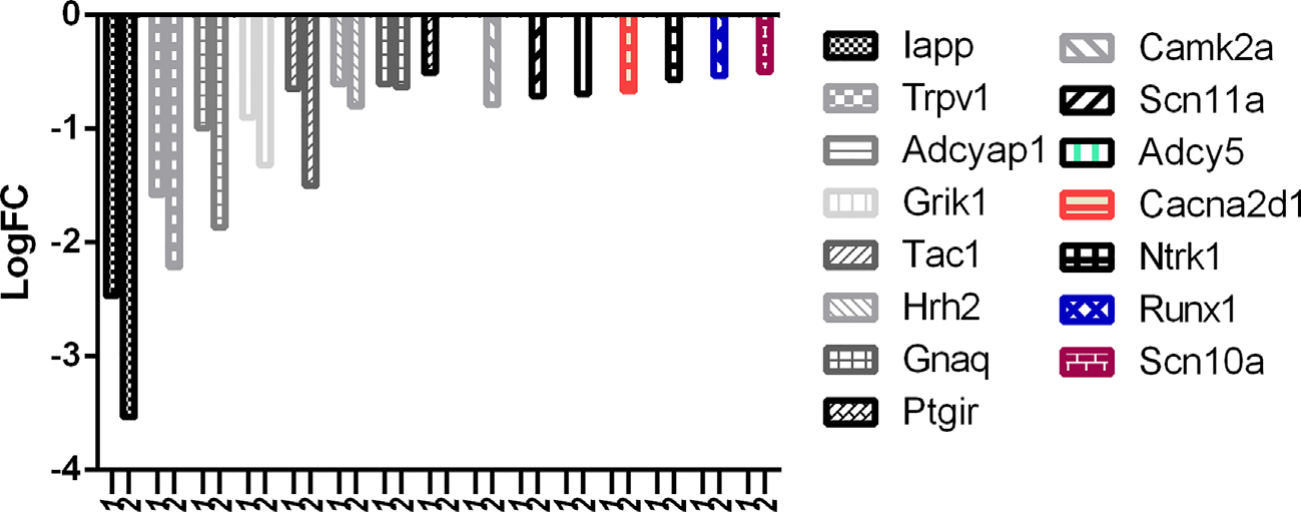
Downregulation of pain genes responsible for increased nociception. 1: capsaicin; 2: RTX.

There is no question about the potency of RTX, but selectivity should also be considered and a balance of both is what required at the clinical level. With reference to **Figure 8**, it can be easily observed that the downregulation of desired pain genes affecting nociception like Iapp, Trpv1, Adcyap1, Grik1, Tac1, Hrh2, and Gnaq was more evident in the RTX-treated group when compared to the capsaicin-treated group. Interestingly, only capsaicin was able to downregulate Ptgir, while only RTX was able to downregulate some other desired pain genes like Camk2a, Scn11a, Adcy5, Cacna2d1, Ntrk1, Runx1, and Scn10a.

**Figure 9:**
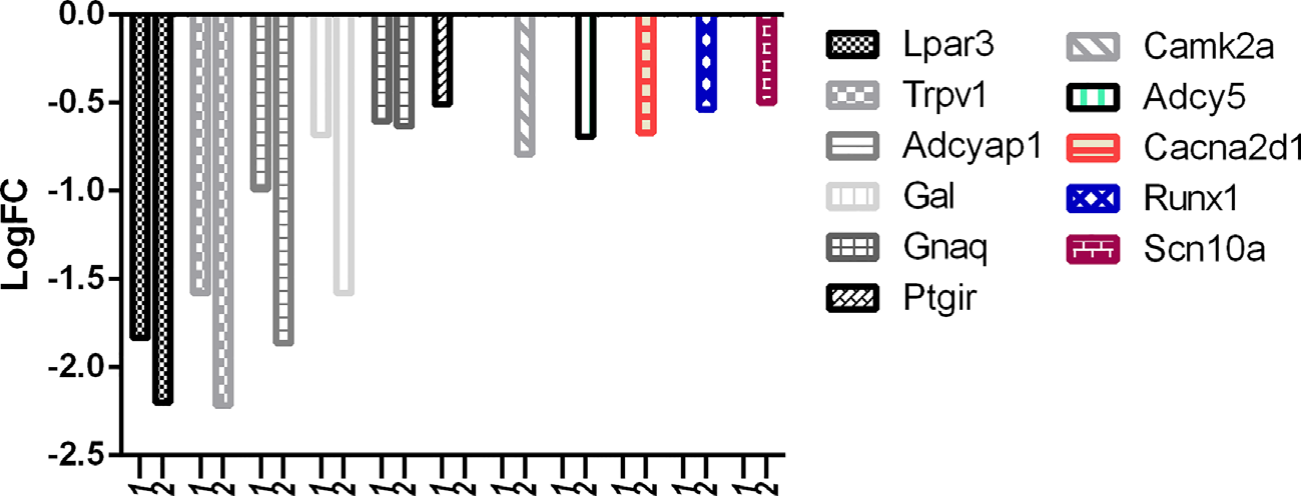
Downregulation of pain genes responsible for increased hypersensitivity. 1: capsaicin; 2: RTX.

With reference to **Figure 9**, it is evident that the downregulation of desired pain genes affecting hypersensitivity like Lpar3, Trpv1, Adcyap1, Gal, and Gnaq was observed more in the RTX-treated group when compared to the capsaicin-treated group. Similarly, only capsaicin was able to downregulate Ptgir while only RTX was able to downregulate Camk2a, Adcy5, Cacna2d1, Runx1, and Scn10a.

**Figure 10:**
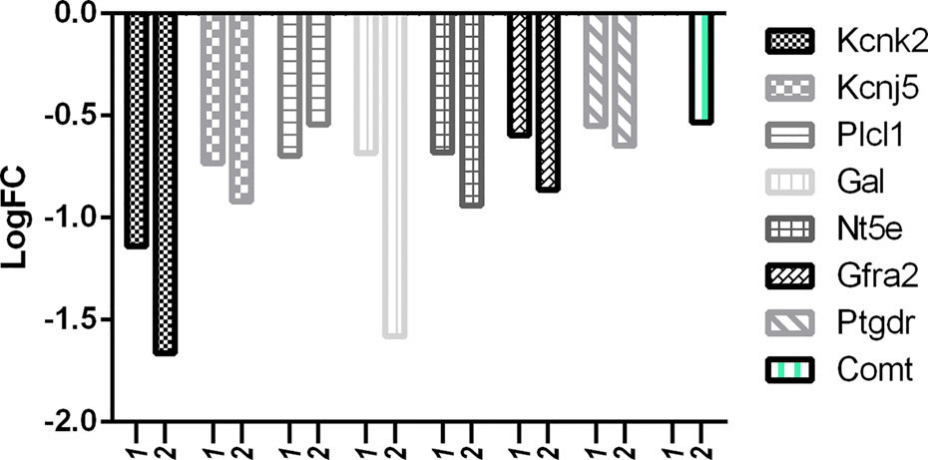
Downregulation of pain genes responsible for decreased nociception. 1: capsaicin; 2: RTX.

**Figure 11:**
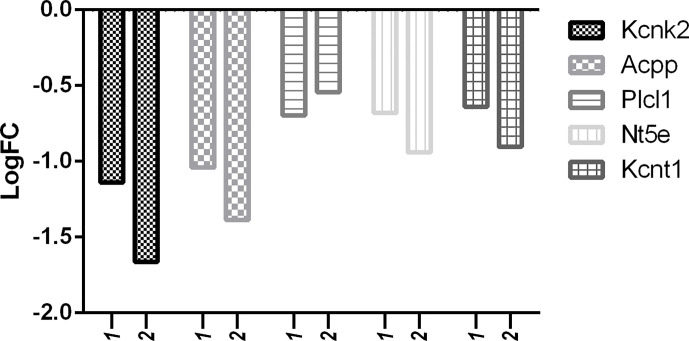
Downregulation of pain genes responsible for decreased hypersensitivity. 1: capsaicin; 2: RTX.

But in comparison with capsaicin, RTX was not only able to downregulate more of the target pain genes, but also more of the off-target pain genes. With reference to **Figure 10**, it is clear that the downregulation of off-target pain genes associated with nociception like Kcnk2, Kcnj5, Gal, Nt5e, Gfra2, and Ptgdr was higher in RTX when compared to capsaicin, except Plcl1 which was downregulated more by capsaicin. Further, only RTX could downregulate the Comt gene. With reference to **Figure 11**, it can be seen that the downregulation of off-target pain genes associated with hypersensitivity like Kcnk2, Acpp, Nt5e, and Kcnt1 was more evident RTX when compared to capsaicin, except Plcl1 which was downregulated more by capsaicin.

So, after reassessing the transcriptomic data, we have found that when compared to capsaicin, RTX not only upregulates more non-pain related genes, but also downregulates more undesired/off-targets pain genes. This proposes that RTX is a more potent drug compared to capsaicin for its clinical implications.

## Data Availability Statement

The datasets presented in this study can be found in online repositories. The names of the repository/repositories and accession number(s) can be found below: https://www.ncbi.nlm.nih.gov/genbank/, GSE59727.

## Author Contributions

RS and BS designed the experiment. RS, AS, and MS participated in data analysis, All authors contributed to the article and approved the submitted version.

## Funding

This work was supported by the National Key Research and Development Program of China (2016YFC1306605) and the National Natural Science Foundation of China (Grant Nos. 31670851).

## Conflict of Interest

The authors declare that the research was conducted in the absence of any commercial or financial relationships that could be construed as a potential conflict of interest.
