# Supplementary material for: Regulation of Pain Genes—Capsaicin vs Resiniferatoxin: Reassessment of Transcriptomic Data
Source: Front Pharmacol. 2020 Oct 29;11:551786. doi: 10.3389/fphar.2020.551786 (PMC7658921; doi:10.3389/fphar.2020.551786)
Supplement: Supplementary file 1 [file DataSheet_1.docx]

**Table S1: Top 30 Up-regulated and down-regulated genes for Capsaicin (10µM)**

| **ID** | **P- Value** | **logFC** | **Gene**  **symbol** | **Expression** | **P- Value** | **logFC** | **Gene**  **symbol** | **Expression** |
| --- | --- | --- | --- | --- | --- | --- | --- | --- |
| 1 | 0.0002 | 0.6969 | Olr1349 | Up | 2.39E-05 | -0.64070 | Celf6 | Down |
| 2 | 0.0003 | 0.6598 | Olr190 | Up | 3.13E-05 | -0.6509 | Dapk1 | Down |
| 3 | 0.0016 | 0.6580 | Tmprss5 | Up | 4.22E-05 | -0.6962 | Plcl1 | Down |
| 4 | 0.0033 | 0.6493 | Notch3 | Up | 2.39E-05 | -0.7021 | Chn1 | Down |
| 5 | 0.0023 | 0.6462 | Ctss | Up | 4.48E-05 | -0.7034 | Galk1 | Down |
| 6 | 0.0019 | 0.6349 | Ccl4 | Up | 1.07E-05 | -0.7112 | Scml4 | Down |
| 7 | 0.0002 | 0.6237 | Car13 | Up | 3.27E-05 | -0.7404 | Galr2 | Down |
| 8 | 0.0025 | 0.6211 | Napsa | Up | 2.41E-05 | -0.7595 | B3galnt1 | Down |
| 9 | 0.0004 | 0.6164 | Colec12 | Up | 2.60E-05 | -0.7949 | Ina | Down |
| 10 | 0.0025 | 0.5950 | Eif2a | Up | 3.31E-06 | -0.8612 | Rgs4 | Down |
| 11 | 0.0017 | 0.5844 | Ns5atp9 | Up | 1.45E-05 | -0.8635 | Dpysl4 | Down |
| 12 | 0.0013 | 0.5785 | Helz2 | Up | 8.62E-06 | -0.8796 | Cdh11 | Down |
| 13 | 0.0007 | 0.5750 | Ackr3 | Up | 5.43E-06 | -0.9037 | Grik1 | Down |
| 14 | 0.0009 | 0.5684 | Gmfg | Up | 2.54E-06 | -0.9641 | Kcnip2 | Down |
| 15 | 0.0024 | 0.5673 | Aif1 | Up | 1.57E-06 | -0.9912 | Adcyap1 | Down |
| 16 | 0.0008 | 0.5639 | Btk | Up | 1.62E-06 | -1.0417 | Acpp | Down |
| 17 | 0.0024 | 0.5624 | Tbxas1 | Up | 3.48E-05 | -1.0467 | Amigo2 | Down |
| 18 | 0.0010 | 0.5616 | Rrad | Up | 2.79E-06 | -1.0740 | Nnat | Down |
| 19 | 0.0004 | 0.5573 | Fbln5 | Up | 3.64E-05 | -1.1393 | Kcnk2 | Down |
| 20 | 0.0013 | 0.5524 | Plscr1 | Up | 4.69E-07 | -1.1521 | Gfra3 | Down |
| 21 | 0.0001 | 0.5508 | S100a16 | Up | 2.28E-05 | -1.1924 | Trpc3 | Down |
| 22 | 0.0008 | 0.5463 | Mrgprb4 | Up | 1.68E-05 | -1.3871 | Mrgprd | Down |
| 23 | 0.0012 | 0.5430 | Arhgdib | Up | 7.90E-07 | -1.4534 | Amdhd1 | Down |
| 24 | 0.0029 | 0.5427 | Cp | Up | 4.97E-07 | -1.4553 | Slc51a | Down |
| 25 | 0.0015 | 0.5400 | Unc5cl | Up | 2.54E-08 | -1.5814 | Trpv1 | Down |
| 26 | 0.0011 | 0.5376 | Sst | Up | 1.36E-05 | -1.8342 | Lpar3 | Down |
| 27 | 0.0032 | 0.5370 | Cd52 | Up | 2.71E-06 | -2.0319 | Kcnip1 | Down |
| 28 | 0.0006 | 0.5366 | Hectd2 | Up | 1.74E-08 | -2.1140 | Mrgprx3 | Down |
| 29 | 0.0004 | 0.5141 | Pltp | Up | 6.23E-09 | -2.4697 | Iapp | Down |
| 30 | 0.0028 | 0.5091 | Nmu | Up | 7.39E-08 | -2.9376 | Cartpt | Down |

**Table S2: Top 30 Up-regulated and down-regulated genes for RTX (100nM)**

| **ID** | **P-Value** | **logFC** | **Gene**  **symbol** | **Expression** | **P-Value** | **logFC** | **Gene**  **symbol** | **Expression** |
| --- | --- | --- | --- | --- | --- | --- | --- | --- |
| 1 | 0.0003 | 0.7712 | Notch3 | Up | 1.39E-05 | -0.7527 | Paqr5 | Down |
| 2 | 0.0004 | 0.7302 | Itga5 | Up | 9.84E-06 | -0.8431 | Dapk1 | Down |
| 3 | 0.0008 | 0.7175 | Traf3ip3 | Up | 7.44E-06 | -0.8521 | Scml4 | Down |
| 4 | 9.83E-05 | 0.6871 | Hpse | Up | 1.42E-05 | -0.8558 | Cdh11 | Down |
| 5 | 0.0006 | 0.6572 | Mylip | Up | 8.70E-06 | -0.8639 | Gfra2 | Down |
| 6 | 0.0006 | 0.6567 | Pxn | Up | 1.31E-05 | -0.8947 | Dpysl4 | Down |
| 7 | 0.0003 | 0.6500 | Aoc3 | Up | 8.23E-06 | -0.9040 | Kcnt1 | Down |
| 8 | 0.0004 | 0.6460 | Plpp3 | Up | 5.50E-06 | -0.9229 | Galk1 | Down |
| 9 | 0.0001 | 0.6289 | Bace2 | Up | 2.59E-06 | -1.0940 | Kcng1 | Down |
| 10 | 0.0001 | 0.6133 | S100a16 | Up | 7.23E-07 | -1.0943 | Kcnip2 | Down |
| 11 | 0.0003 | 0.6130 | S100b | Up | 8.69E-06 | -1.1818 | Hs6st2 | Down |
| 12 | 0.0003 | 0.5886 | Tnfaip8 | Up | 1.80E-07 | -1.1870 | Rgs4 | Down |
| 13 | 0.0001 | 0.5842 | Gpr37l1 | Up | 1.13E-06 | -1.2025 | Galr2 | Down |
| 14 | 0.0002 | 0.5840 | Adamts9 | Up | 2.26E-07 | -1.3182 | Grik1 | Down |
| 15 | 0.0005 | 0.5822 | Fbln5 | Up | 6.31E-07 | -1.3861 | Acpp | Down |
| 16 | 0.0005 | 0.5668 | Lhfp | Up | 1.60E-06 | -1.4753 | Trpc3 | Down |
| 17 | 0.0009 | 0.5553 | Itgb5 | Up | 3.77E-07 | -1.5033 | Tac1 | Down |
| 18 | 0.0004 | 0.5542 | Anxa4 | Up | 2.98E-06 | -1.5818 | Gal | Down |
| 19 | 0.0003 | 0.5507 | Spp1 | Up | 4.76E-07 | -1.6639 | Kcnk2 | Down |
| 20 | 0.0002 | 0.5501 | Lamb2 | Up | 3.31E-08 | -1.7723 | Amdhd1 | Down |
| 21 | 0.0008 | 0.5486 | Chst1 | Up | 1.09E-05 | -1.7864 | Mrgprd | Down |
| 22 | 0.0003 | 0.5412 | Vsnl1 | Up | 2.03E-08 | -1.8648 | Adcyap1 | Down |
| 23 | 0.0004 | 0.5367 | Plat | Up | 1.60E-09 | -2.1022 | Slc51a | Down |
| 24 | 0.0002 | 0.5343 | Slc13a3 | Up | 6.29E-07 | -2.1247 | Gfra3 | Down |
| 25 | 0.0007 | 0.5330 | Cdc42ep1 | Up | 1.41E-06 | -2.1980 | Lpar3 | Down |
| 26 | 0.0008 | 0.5311 | Pon2 | Up | 9.10E-10 | -2.2152 | Trpv1 | Down |
| 27 | 0.0005 | 0.5284 | Plekhb1 | Up | 8.08E-10 | -2.7634 | Mrgprx3 | Down |
| 28 | 0.0004 | 0.5077 | Pgf | Up | 2.03E-06 | -2.9989 | Crh | Down |
| 29 | 0.0005 | 0.5058 | Bhlhe40 | Up | 3.97E-08 | -3.4566 | Cartpt | Down |
| 30 | 0.0008 | 0.5031 | Sparcl1 | Up | 1.00E-07 | -3.5366 | Iapp | Down |
