# Supplementary material for: Regulation of Pain Genes—Capsaicin vs Resiniferatoxin: Reassessment of Transcriptomic Data
Source: Front Pharmacol. 2020 Oct 29;11:551786. doi: 10.3389/fphar.2020.551786 (PMC7658921; doi:10.3389/fphar.2020.551786)
Supplement: Supplementary file 2 [file Presentation_1.pptx]

## Slide 1
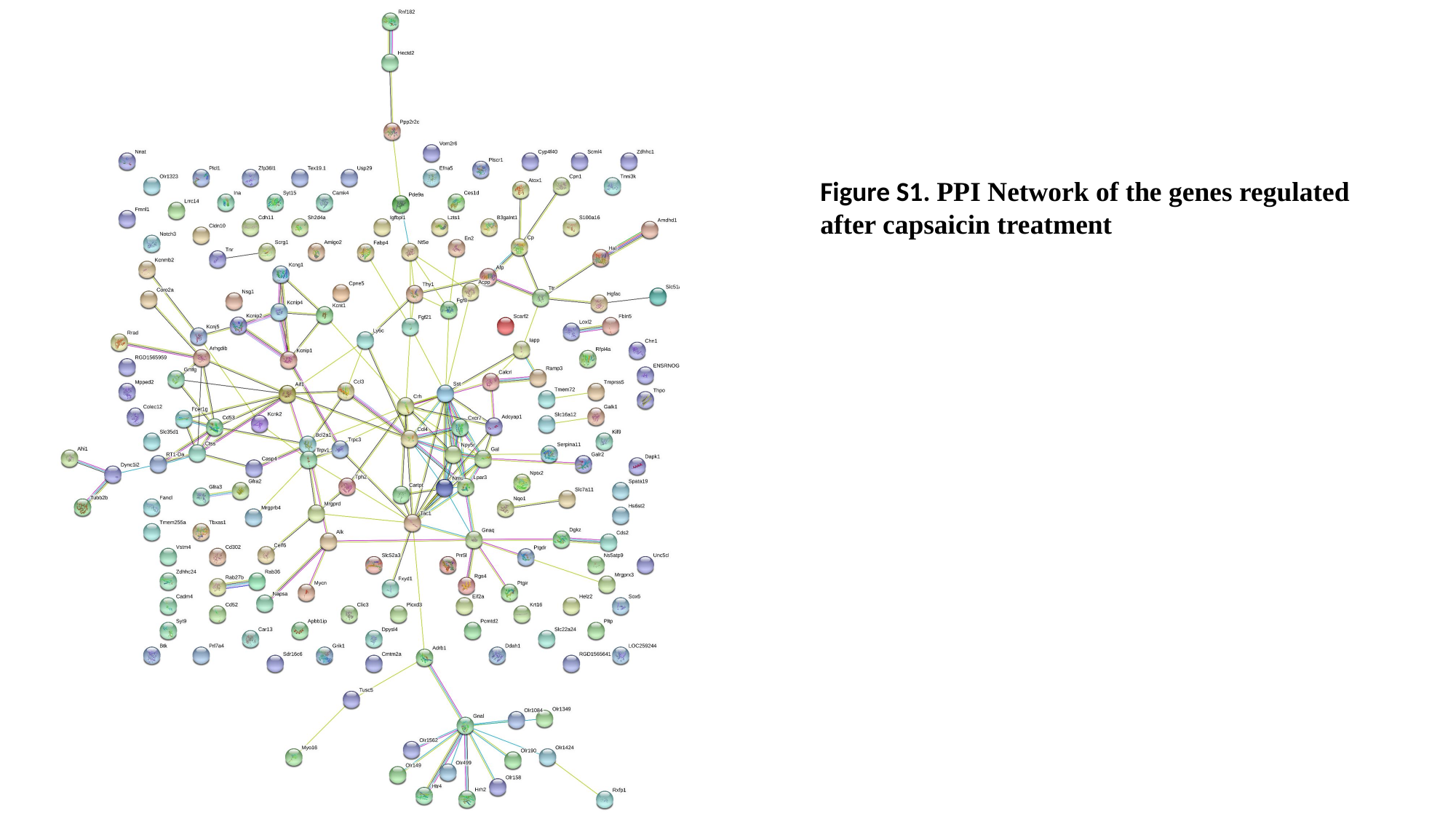

Figure S1. PPI Network of the genes regulated after capsaicin treatment

## Slide 2
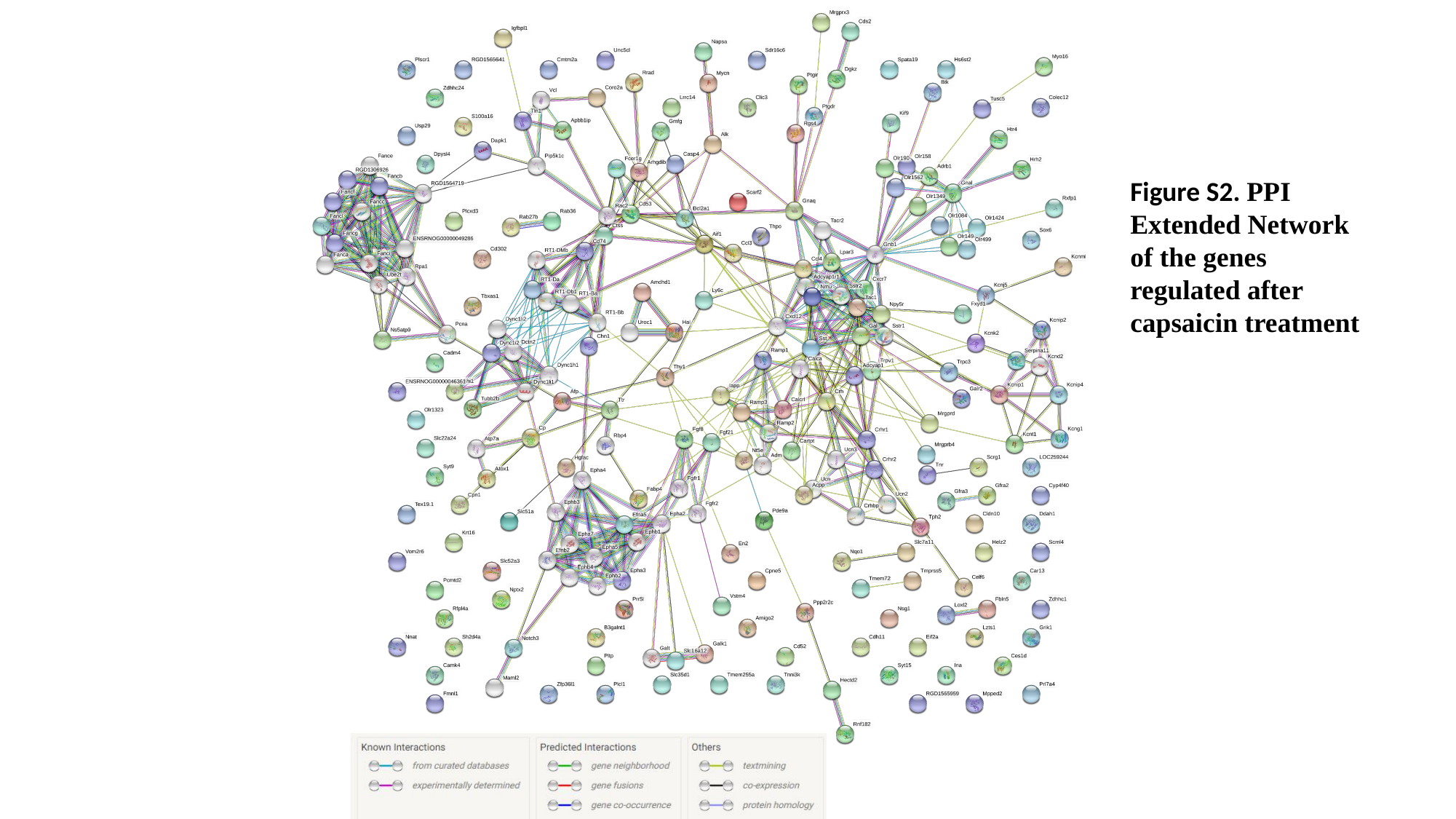

Figure S2. PPI Extended Network of the genes regulated after capsaicin treatment

## Slide 3
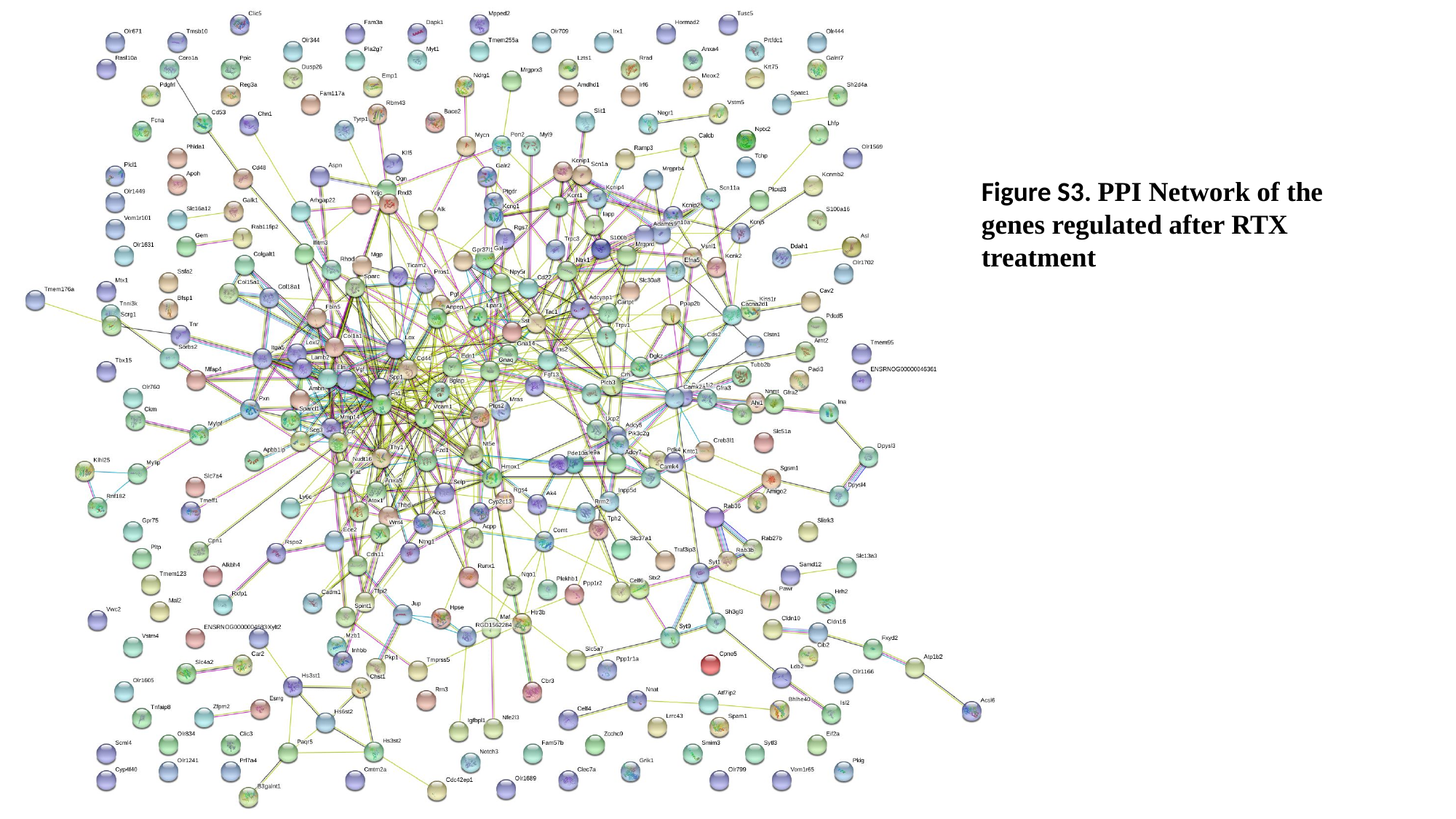

Figure S3. PPI Network of the genes regulated after RTX treatment

## Slide 4
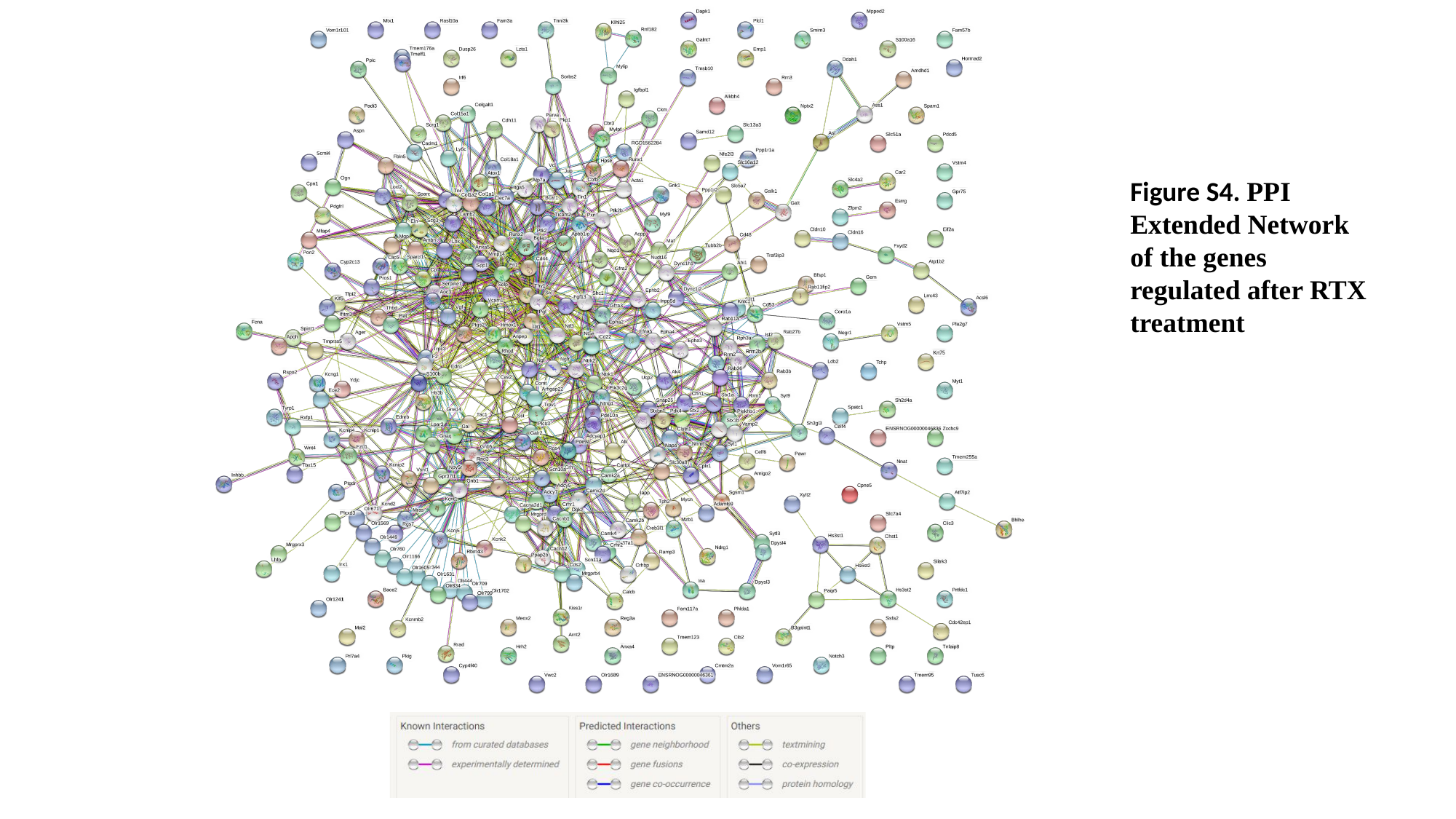

Figure S4. PPI Extended Network of the genes regulated after RTX treatment
